# Integrated lipidomics and transcriptomic analysis of peripheral blood reveals significantly enriched pathways in type 2 diabetes mellitus

**DOI:** 10.1186/1755-8794-6-S1-S12

**Published:** 2013-01-23

**Authors:** Chen Zhao, Jinghe Mao, Junmei Ai, Ming Shenwu, Tieliu Shi, Daqing Zhang, Xiaonan Wang, Yunliang Wang, Youping Deng

**Affiliations:** 1 Wuhan University of Science and Technology, Wuhan, Hubei 430081, P.R. China; 2 Center for Bioinformatics and Computational Biology, Shanghai Key Laboratory of Regulatory Biology, the Institute of Biomedical Sciences and School of Life Science, East China Normal University, Shanghai 200241, China; 3 Department of Biology, Tougaloo College, Tougaloo, MS 39174, USA; 4 Department of Internal Medicine, Rush University Cancer Center, Rush University Medical Center, Chicago, IL 60612, USA; 5 Center for Systems Biology, School of Mathematical Sciences, Soochow University, Suzhou, Jiangsu 215006, China; 6 Department of Neurology, The 148 Hospital of PLA, Zibo, Shandong, 255300, China

## Abstract

**Background:**

Insulin resistance is a key element in the pathogenesis of type 2 diabetes mellitus. Plasma free fatty acids were assumed to mediate the insulin resistance, while the relationship between lipid and glucose disposal remains to be demonstrated across liver, skeletal muscle and blood.

**Methods:**

We profiled both lipidomics and gene expression of 144 total peripheral blood samples, 84 from patients with T2D and 60 from healthy controls. Then, factor and partial least squares models were used to perform a combined analysis of lipidomics and gene expression profiles to uncover the bioprocesses that are associated with lipidomic profiles in type 2 diabetes.

**Results:**

According to factor analysis of the lipidomic profile, several species of lipids were found to be correlated with different phenotypes, including diabetes-related C23:2CE, C23:3CE, C23:4CE, ePE36:4, ePE36:5, ePE36:6; race-related (African-American) PI36:1; and sex-related PE34:1 and LPC18:2. The major variance of gene expression profile was not caused by known factors and no significant difference can be directly derived from differential gene expression profile. However, the combination of lipidomic and gene expression analyses allows us to reveal the correlation between the altered lipid profile with significantly enriched pathways, such as one carbon pool by folate, arachidonic acid metabolism, insulin signaling pathway, amino sugar and nucleotide sugar metabolism, propanoate metabolism, and starch and sucrose metabolism. The genes in these pathways showed a good capability to classify diabetes samples.

**Conclusion:**

Combined analysis of gene expression and lipidomic profiling reveals type 2 diabetes-associated lipid species and enriched biological pathways in peripheral blood, while gene expression profile does not show direct correlation. Our findings provide a new clue to better understand the mechanism of disordered lipid metabolism in association with type 2 diabetes.

## Background

Skeletal muscle and hepatic insulin resistance are key elements in the pathogenesis of type 2 diabetes mellitus (T2D) [1]. However, T2D is caused by not only insulin resistance [2], but also a heterogeneous cluster of conditions rather than a uniform entity [3]. Due to both environment and heredity heterogeneity, gene expression profiling is limited in exploring molecular mechanism of type 2 diabetes [4,5].

As a comprehensive indicator, plasma free fatty acids were assumed to mediate the insulin resistance. Lipid profiling has already been applied in type 2 diabetes studies [6,7], such as free fatty acids built linkage between the resistance and obesity [8]. However, the relationship between lipid and glucose disposal remains to be demonstrated across liver, skeletal muscle, and blood [9,10]. Here, we have integrated lipidomic analysis with gene expression profiling to discover the relationship between versatile lipid species and bioprocesses that are associated with type 2 diabetes. Using our model analysis, the statistically significant biological pathways were retrieved, and the findings provide a new strategy to link blood lipid species and illuminate the mechanism of insulin resistance associated with lipid and gene expression in blood.

## Results

### Study subjects

This study comprised a balanced distribution of the studied subjects in gender and race: among 60 controls, 28 were African American (AA) including 14 females and 14 males; 32 were Caucasian (CAU) including 14 females and 18 males. Among 84 patients with T2D, 44 were AA including 22 females and 22 males; 40 were CAU including 23 females and 17 males. As compared to AA, CAU had a significantly higher level of blood triglycerides (TG) in both the controls (106 ± 54.3 mg/dl in AA vs. 153 ± 77.8 mg/dl in CAU, p = 0.0009), and the patients (157 ± 128 mg/dl in AA vs. 207 ± 98.3 mg/dl in CAU, p = 0.037). There were no significant differences in other studied clinical parameters between two races (data for racial differences were not shown). As compared to all controls (mixed), patient's group was 4.5 years older, had significantly higher body mass index (BMI), blood TG and fasting glucose, and lower high density apolipoprotein (HDL). There were no differences in low density apolipoproteins (LDL) and total cholesterols (Table 1) between controls and T2D patients.

**Table 1:** The clinical characteristics of the study subjects

	Normal controls (*n *= 60)	Diabetic (*n *= 84)
Age, yr (mean ± SD)	58.5 ± 16.1	63 ± 13
Sex (female/male)	28/32	45/39
Race	32 Caucasian	40 Caucasian
	28 African American	44 African American
Body Mass index (kg/m)	30.1 ± 7.3	34.2 ± 8.4**
Triacylglycerol (mg/dl)	134 ± 76.9	186 ± 113.8**
HDL cholesterol (mg/dl)	56.6 ± 17.5	49.1 ± 15.4**
LDL cholesterol (mg/dl)	112 ± 44.8	109.8 ± 36.5
Total cholesterol (mg/dl)	197 ± 44.8	195 ± 46.8
Glucose (mg/dl)	88.8 ± 10.8	142.7 ± 56.8***

Data are mean ± SD. Statistical analyses were performed using the Student's t-Test. **p < 0.05 ***p < 0.001, values are based on differences from controls.

### Plasma lipid profile reveals phenotype factors

**Figure 1:**
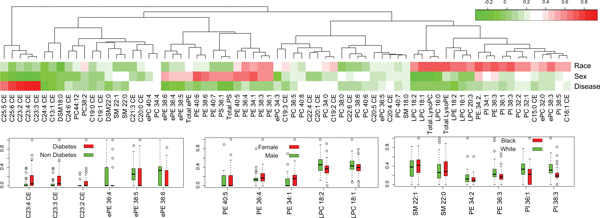
**Factor analysis of lipidomic profile**. The upper panel is a heatmap of factory analysis. Factor loadings, where race, sex, and disease correspond to the three factors. Color depth represents for factor loadings of 71 different lipid indicators, positive loading were shown in red and negative loadings in green. The lower panel is a boxplot of the important loading lipid for three known factor, including diabetes, sex and race. The lipid levels were scaled to the range 0 to 1, and each lipid corresponds to two boxes with different factor levels.

Plasma lipid profile is associated with various types of diseases or phenotypes. In order to illustrate the relationship between lipid species and gene expression level of peripheral blood, we performed unsupervised exploratory factor analysis and found significant linkages between lipid profile and phenotypes, including race, sex, and diabetes at the significant levels 1.87e-6, 9.28e-4, and 3.17e-3 by Wilcoxon Rank Sum Test, respectively. As shown in Figure 1, three types of CE species (C23:2CE, C23:3CE, C23:4CE) were found to be positively correlated with diabetes, while three types of ePE were shown to be negatively correlated. For sex, more than five and six lipid species were found to be correlated: PE40:5, PE36.4, and PE34.1 tend to be higher in female samples, while LPC18:2 and LCP18:1 were a little higher in male samples. For race, two types of SM (SM22:1 and SM22:0) were a little higher in black, while PE (PE34:2, PE36:3) and PI (PI36:1, PI38:3) were higher in white samples.

### Phenotype factors have lesser effect on gene expression profile

**Figure 2:**
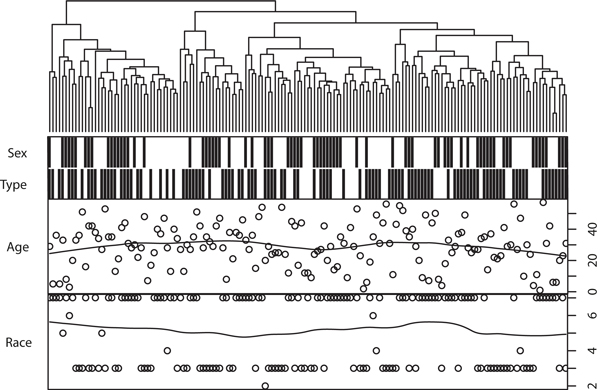
**Hierarchical clustering of all samples in filtered data set**. Factors such as sex and type were represented by black or white blocks: female was in white, male in black; diabetes in black, non-diabetes in white; Asian as 2, blacks as 3, Indian as 4, Mexican as 5 and 6, whites as 7.

Unlike the lipid profile, the gene expression profile does not show direct correlation with phenotype indicators, according to both a hierarchical clustering (Figure 2) and principal component analysis (PCA). As shown in the clustering, all of the data can be divided in to four main classes, but none of the factors (sex, diabetes, age, and race) were significantly correlated with main classes. However, race and sex were shown to be non-randomly distributed in the dendrogram, which implies underlying correlation with gene expression profile (GEP). Moreover, significant correlation was identified between GEP and phenotype factors based on PCA scores in the correlation test. GEP was correlated with race, and many genes may be differentially expressed between black and white samples. Race was the factor most known to be GEP-correlated, and tested as correlated with the third component (p = 7.20e-4, Kruskal test), which contains 5.8% variances. Diabetes was then tested to be correlated with the fifth component (p = 8.67e-3, Kruskal test), which contains 4.7% variances, and sex with the tenth component (p = 2.02e-2, Kruskal test) containing 2.1% variances. There is almost 87.4% variance or unknown information in GEP. Direct differential expression genes were difficult to understanding in terms of biological meanings, which enriched in seemingly unrelated pathways (Table 2) such as ECM-receptor interaction and Riboflavin metabolism.

**Table 2:** Enriched pathways of differentially expressed genes.

KEGGID	P-value	Odds ratio	ExpCount	Count	Size	Term
**4510**	2.95E-05	5.284093	2.478357	11	200	Focal adhesion
**4512**	7.66E-05	7.804973	1.053302	7	85	ECM-receptor interaction
**740**	2.81E-04	31.125	0.13631	3	11	Riboflavin metabolism
**5146**	9.78E-03	4.162056	1.313529	5	106	Amoebiasis

P-value is the significant level of KEGG pathway enrichment based on fisher exact test [16]. Odds Ratio and ExpCount are the corresponding Odds Ratio and expect observed number. Count is the number of differentially expressed genes in corresponding pathway, and size is the total gene number.

### Significant biological pathways link gene expression profile with lipid profile and diabetes

To overcome the limitation of the unknown variances in gene expression profile, and to recover the relationship between gene expression profile and lipid profile, PLS regression model was adopted. A list of significant pathways from the gene expression profile was found to explain the lipid profiles, and also the lipid profile associated T2D (Table 3). Six of the top ten pathways have direct linkage with diabetes, including one carbon pool by folate, arachidonic acid metabolism, insulin signaling pathway, amino sugar and nucleotide sugar metabolism, propanoate metabolism, and starch and sucrose metabolism. None of them can be retrieved from a differential expression gene selection.

**Table 3:** Enriched pathways of differentially expressed genes

Rank	KEGG Path ID	KEGG pathway name	Top 5 loadings gene
**1**	path:hsa00670	One carbon pool by folate	"**MTHFD2L**" "**ALDH1L1**" "MTFMT" "ALDH1L2" "MTR"
**2**	path:hsa00590	Arachidonic acid metabolism	"**PTGS2**""GPX7" "PLB1" "CYP4A11""GPX2"
**3**	path:hsa04910	Insulin signaling pathway	"FLOT2" "PRKAB2""**MAPK8**" "**PPP1R3B**" "PIK3CB"
**4**	path:hsa05110	Vibrio cholerae infection	"TJP2" "ATP6V1C1" "ADCY9""ARF1" "ATP6V1H"
**5**	path:hsa04020	Calcium signaling pathway	"HTR4" "TRPC1""ADCY8""CHRM5""**GRIN2A**"
**6**	path:hsa00520	Amino sugar and nucleotide sugar metabolism	"CHI3L1" "**HK2**""NPL""**HEXA**" "UAP1L1"
**7**	path:hsa04062	Chemokine signaling pathway	"**CXCL5**""CXCL10" "**PF4V1**""**CCL8**" "**CXCL11**"
**8**	path:hsa00640	Propanoate metabolism	"MLYCD" "ALDH2" "ACSS1" "PCCB""ALDH3A2"
**9**	path:hsa00500	Starch and sucrose metabolism	"UGT2B17" "UGT2B15" "MGAM""HK1" "**AMY2B**"
**10**	path:hsa00240	Pyrimidine metabolism	"DPYD""ENTPD1""TXNRD1""TK2" "**TYMP**"

Top Ten Enriched Pathways of most Factor Loading Genes. KEGG Path ID represents for KEGG pathway ID[ 19], which is ranked by pvalues of enrichment analysis. Pathways in gray lines have at least one bold marked gene, which is selected as a feature in gene based classifier. And the pathways in gray lines are the pathways that directly linked with diabetes r by the marked genes.

## Discussion

Gene expression profiling was generally adopted for diabetes in the levels of cell lines and drug response [11,12]. Considering the environment and heredity heterogeneity, the homogeneity is not easy to conclude from a snapshot of the transcriptome for a wide cohort. Thus, we take lipid as an assistant to guide the exploration of gene-level mechanism of insulin resistance associated with lipid and gene expression in blood.

As expected, a major finding in our study is that very limited variance of transcriptome can be illustrated by the known phenotype factors. However, lipid profile shows an unexpected capacity on revealing the considered phenotype factors. By a lipid-guided exploration, a set of significant biological pathways and suspected genes were identified to be insulin resistance-associated, including one carbon pool by folate, arachidonic acid metabolism, and insulin signaling pathway, which cannot be directly found by gene expression profile. Our findings may prompt the understanding of the lipid associated gene-level mechanism of insulin resistance of type 2 diabetes mellitus in blood.

## Materials and methods

### Subjects and clinical laboratory data

The study was approved by the Institutional Review Board of Tougaloo College. All subjects provided written informed consent for this study. T2D was diagnosed based on American Diabetes Association (ADA) [5] and characteristic symptoms of diabetes, a higher BMI, and a fasting plasma glucose > 126 mg dl^-1 ^or a 2 h plasma glucose during an oral glucose tolerance test of > 200 mg dl^-1^. A total of 144 blood samples from healthy controls (*n *= 60, 32 Caucasians and 28 African Americans), and T2D (*n *= 84, 40 Caucasians and 44 African Americans) were collected. All subjects were evaluated by age, sex, race, body mass index (BMI), triacylglycerol (TG), high-density lipoprotein (HDL), low-density lipoprotein (LDL), total cholesterol (TC), and glucose levels.

### Microarray experiments

Total RNA from 8-10 mls peripheral blood WBCs was obtained using LeukoLock™ Total RNA system (Ambion Inc, Austin, TX) according to the manufacturer's instructions. The quantity and quality of the isolated RNA were evaluated by Nanodrop spectrophotometry and Agilent 2100 Bioanalyzer (Agilent Technologies, Santa Clara, CA). Gene expression profiling was peerformed using Agilent Whole Human Genome1 (4 X44K) Oligo arrays with ~20,000 genes represented (Agilent Technologies, Palo Alto, CA). Each sample was hybridized with a human universal RNA control (Stratagene, La Jolla, CA). 500 ng of total RNA was amplified and labeled using the Agilent Low RNA Input Fluorescent Linear Amplification Kit, according to manufacturer's protocol. For each two color array, 850 ng of each *Cy5*- (universal control) and *Cy3*-labeled (sample) cRNA were mixed and fragmented using the Agilent *In Situ *Hybridization Kit protocol. Hybridizations were performed for 17 hours in a rotating hybridization oven according to the Agilent 60-mer oligo microarray processing protocol prior to washing and scanning with an Agilent Scanner (G2565AA, Agilent Technologies, Wilmington, DE). Arrays were processed and background corrected with default settings for all parameters with the Agilent Feature Extraction software (v.9.5.3.1).

### Microarray data analysis

Microarray data analyses were processed with GeneSpring version 7.0 and 10.0. The sample quality control was based on the Pearson correlation of a sample with other samples in the whole experiment. If the average Pearson correlation with other samples was less than 80%, the sample was excluded for further analysis. More detailed analysis was done similar to previous description [13].

### ESI-MS/MS lipid profiling

The same subjects that used for microarray experiments were also used for lipid profiling. Plasma was directly used for the lipid profiling, which was conducted as described previously [14].

### Statistical analyses

To evaluate the correlation between various type of data and phenotypes, two-side Kruskal's test were performed in R [15]. Pathway analysis of the expression data was performed by Fisher exact test with GOstats [16] package. Factor analyses of lipid profile were also preformed in R, where varimax rotation was used to seek a basis that most economically represents each individual. Feature selection and cSVM classifier were implement with CMA [17]. PLS regression model were built [18] with leave-one-out cross-validation.

## Competing interests

The authors declare that they have no competing interests.

## Authors' contributions

YD initiated and oversaw the project. YD and MJ designed the study. MJ and MS conducted the experiments. YD. JA, ST, DZ and ZC performed the data analysis. YD and ZC drafted the manuscript. YD, ZC, DZ, XW and TS contributed to result interpretation and manuscript preparation.
